# Antifungal Activity and Composition of Essential Oils of *Conyza canadensis* Herbs and Roots

**DOI:** 10.1100/2012/489646

**Published:** 2012-05-01

**Authors:** Katalin Veres, Boglárka Csupor-Löffler, Andrea Lázár, Judit Hohmann

**Affiliations:** ^1^Department of Pharmacognosy, University of Szeged, Eötvös u. 6, 6720 Szeged, Hungary; ^2^Institute of Clinical Microbiology, University of Szeged, Semmelweis u. 6. 6725 Szeged, Hungary

## Abstract

Essential oils from herbs and roots of *Conyza canadensis* (horseweed), collected in Hungary, were obtained by hydrodistillation. The chemical compositions of the oils were analysed by combination of GC and GC/MS. The major constituent of the oil obtained from the aerial parts of horseweed was limonene (78%), while the main component of root oil was 2*Z*,8*Z*-matricaria ester. The antimicrobial activities of the oils were tested on Gram-positive (*Enterococcus faecalis*, *Staphylococcus aureus*, and *Streptococcus pyogenes*), Gram-negative (*Escherichia coli, Pseudomonas aeruginosa*) bacteria, reference fungal strains, and fungal strains isolated from patients (*Candida*, *Cryptococcus*, *Trichophyton*, *Rhodotorula*, and *Aspergillus*) by agar disc diffusion and broth dilution methods. None of the oils showed any activity against the tested bacterial strains, but exhibited moderate-to-strong activity against all fungi with the only exception of *A. fumigatus*. The highest zone of inhibition was observed in case of *Cryptococcus neoformans* and *Trichophyton interdigitalis*

## 1. Introduction

*Conyza canadensis* (L.) Cronq. (formerly *Erigeron canadensis* L.; horseweed or canadian fleabane, Asteraceae) is indigenous to North America but is globally distributed today and widely found in Hungary. The aerial parts and the roots of this plant have been used all over the world as traditional or official herbal medicines for the treatment of gastrointestinal symptoms, most commonly diarrhoea and dysentery, and as diuretic agent [1]. In Chinese folk medicine, horseweed has also been applied for the treatment of wounds, swellings, and pain caused by arthritis [2]. Moreover, the volatile oil of horseweed has been applied against bronchitis and cystitis [3].

Phytochemical studies of *C. canadensis* revealed the presence of C_10_ acetylenes [4, 5], sesquiterpene hydrocarbons [6], flavonoids [7], sterols, triterpenes, and sphingolipids [8–10]. The essential oil of juvenile and mature herbs collected in Washington State (USA) was previously analyzed by Hrutfiord et al.; twenty-five constituents, including monoterpenes, sesquiterpenes, and acetylenes were identified, and the predominance of limonene (67.25%) was confirmed [11]. In a sample of aerial parts collected in France, 18 compounds were detected with limonene as the main constituent (76.0%) [12]. The analysis of the essential oil of aerial part of horseweed growing in Japan led to the detection of 47 volatile components of which 91.0% were terpenoid. The main constituent was limonene (31.2%). Some nonterpenoid acetylenic compounds were also detected [13].

In the present study the composition of essential oils obtained from roots and herbs of Hungarian population of *C. canadensis *was analyzed, and the antimicrobial activities of the oils against human pathogenic bacteria and fungi were evaluated for the first time.

## 2. Material and Methods

### 2.1. Plant Material

Roots (roots 1) and flowering shoots of *Conyza canadensis* were collected in Szeged (Hungary) in July 2009. Further sample from horseweed roots (roots 2) was gathered in Jakabszállás (Hungary) in September 2009. The comminuted plant materials were dried at room temperature. 85.5 g of the shoots, 94.9 g of the roots 1, and 50.45 g of the roots 2 were used for the hydrodistillation. Voucher specimens (No. 804 and 805) have been deposited at the Department of Pharmacognosy, University of Szeged.

### 2.2. Isolation of the Essential Oil

The dried flowering shoots and roots were cut off and were subjected to hydrodistillation for 2 hours, according to the method of Ph. Eur 6.0 [14]. The obtained essential oils were dried over anhydrous sodium sulphate, filtered, and stored at −18°C. The yields were calculated on the basis of the dry weight of the plant materials. The compositions of the oils were studied by GC and GC-MS techniques.

### 2.3. Gas Chromatography

The GC analysis was carried out with an HP 5890 Series II gas chromatograph (FID), using a 30 m × 0.35 mm × 0.25 *μ*m HP-5 fused silica capillary column. The temperature program was from 60°C to 210°C at 3°C min^−1^ and from 210°C to 250°C (2 min hold) at 5°C min^−1^. The detector and injector temperature was 250°C, and the carrier gas was N_2_, with split sample introduction.

### 2.4. Gas Chromatography-Mass Spectrometry

GC-MS analysis was performed with a FINNIGAN GCQ ion trap bench-top mass spectrometer. All conditions were as above except that the carrier gas was He at a linear velocity of 31.9 cmsec^−1^ and the capillary column was DB*-*5MS (30 m × 0.25 mm × 0.25 *μ*m). Positive ion electron ionization mode was used, with a mass range of 40–400 amu. The constituents were identified by comparing their Rts and Kovats indices with published MS data [15] and from computer library searches. The identification was further confirmed with the aid of authentic samples (Extrasynthese, Genay, France). Kovats indices were calculated mainly from the GC-MS analysis results [16].

### 2.5. Bacteriostatic and Fungistatic Activities

Bacterial strains: *Enterococcus faecalis *(ATCC29212), *Staphylococcus aureus *(ATCC25923), *Streptococcus pyogenes *(HNCMB80002) Gram-positive bacteria; *Escherichia coli *(ATCC25922), *Pseudomonas aeruginosa *(ATCC27853) Gram-negative bacteria. Fungal strains: *Candida albicans* (UK-NEQUAS4661), *Candida glabrata* (ATCC90030), *Candida parapsilosis* (ATCC22019), *Candida tropicalis* (UK-NEQUAS4893), *Cryptococcus neoformans* (INF5855) reference fungal strains, and *Candida kefyr, Rhodotorula glutinis, Trichophyton interdigitalis, Aspergillus fumigatus* fungal strains isolated from patients.

The bacteriostatic and fungistatic activities were determined by agar-diffusion method. Different nutrient agars were used: Mueller-Hinton agar (MHA) for the bacteria strains (except *S. pyogenes*), Blood agar (BA) for the *S. pyogenes* strain, and Mueller-Hinton modified agar (MH-GMB) for *C. neoformans* and Sabouraud dextrose agar (SDA) for the fungal/yeast strains. Agars were distributed to sterilized Petri dishes with a diameter of 9 cm (15 mL). The filter paper discs (6 mm in diameter) were placed onto the agar plates which had previously been inoculated with the tested microorganisms, and then the filter paper discs were individually impregnated with 8 *μ*g of the essential oil. The Petri dishes were kept at 4°C for 30 min and then at room temperature for 30 min. The plates were inoculated with bacteria, incubated at 37°C for 24 h, and for 48 h for the yeasts and fungal strains. The diameters of the inhibition zones were measured in millimetres. Piperacillin/tazobactam (100/10 *μ*g), fluconazole (25 *μ*g), and voriconazole (1 *μ*g) served as positive controls.

The MIC (minimal inhibitory concentration) values were determined with the broth dilution assay. MIC values were measured in sterile 96-well microtiter plates. 100 *μ*L aliquots of blank solvent, positive control, and oil solution (dissolved in DMSO at 20 v/v%) were pipetted into the wells in the 1–3 column of the plate, 50 *μ*L of phosphate-buffered saline (PBS) solution into the wells in columns 4–12. The samples of the oils in the third column were diluted with an 8-channel pipette with 50 *μ*L from the 3rd column to the 12th. The 24 h cultured yeast strains were diluted 100-fold in SDA for the *Candida* strains and 10-fold for the *C. neoformans *and* R. glutinis* strains. From these suspensions, 50 *μ*L was added to each well. The samples were then incubated for 48 hours at room temperature in case of the yeasts. The MIC was defined as the lowest concentration of the test sample that resulted in a complete inhibition of visible growth in the broth. DMSO was used as a negative control, while fluconazole was applied as a positive control.

## 3. Results and Discussion

**Figure 1:**
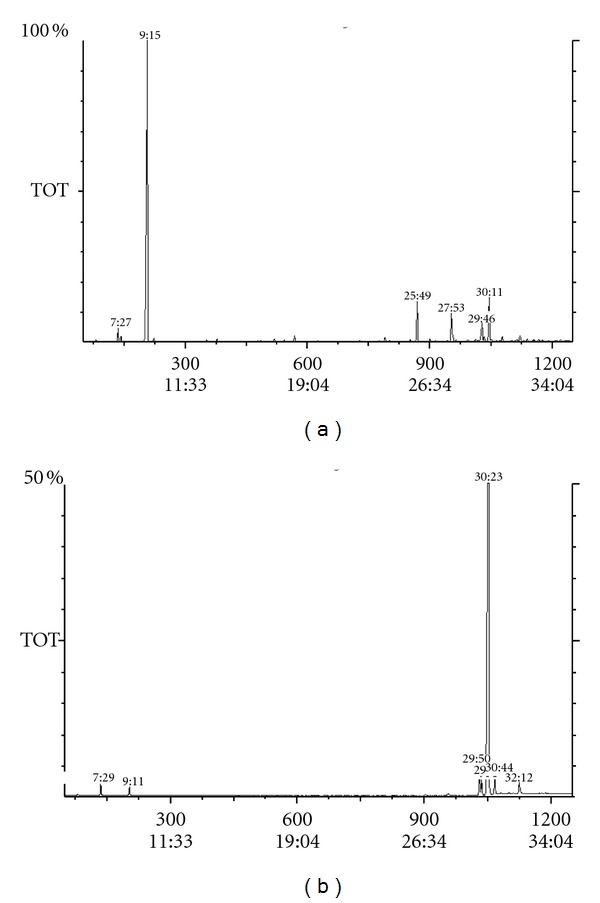
Total ion chromatogram of the essential oil of *Conyza canadensis* (a) oil distilled from the herbs, (b) oil distilled from the roots I.

Essential oils from herbs and two samples of roots of *C. canadensis* were prepared by hydrodistillation. The essential oil content of the roots was much lower (0.20%) than that of the herbs (0.72%). Chemical composition of the oils was analysed by combination of GC and GC/MS (Figure 1) and substantial differences were found between the two investigated plant parts.  Table 1 presents the results of the qualitative and quantitative analyses. The essential oil of the herbs was found to be more complex than the oil of the roots. In the essential oil of the herbs 34 components were detected while in the essential oil of the roots only 9 components could be observed. The identified constituents represented 98.0–99.9% of the oils. The major constituent of the oil of the aerial parts was limonene (79.2%). Further compounds were mono- and sesquiterpenes in 8.6% and 6.6%, and acetylenes in 3.4%. The main component of the oil obtained from the roots was the acetylene compound, 2*Z*,8*Z*-matricaria ester (88.2%; 93.9%), and three other acetylenes (8*Z*-2,3-dihydromatricaria ester, 2*E*,8*Z*-matricaria ester, 4*Z*,8*Z*-matricaria lactone) were present in smaller amounts. Additional differences were observed in the minor components of the two different root samples; *β*-pinene, limonene, 4*E*,8*Z*-matricaria lactone, and 4*Z*,8*Z*-matricaria lactone were found only in roots 1. Interestingly the main compound of the root 2*Z*,8*Z*-matricaria ester can be detected in herb only in small quantity, and, reversed, the main component of the herb limonene is present in the root oil only in 1%.

The antimicrobial activities of oils of the herbs and roots 1 were tested on Gram-positive and Gram-negative bacteria, reference fungal strains, and fungal strains isolated from patients. The agar diffusion method furnished semiquantitative data on the bacteriostatic and fungistatic effects of the oils (Table 2). No substantial differences were found between the activities of the studied essential oils; none of them showed any activity against the tested bacterial strains, but exhibited moderate-to-strong activity against all fungi with the only exception of *Aspergillus fumigatus*. The highest zone of inhibition was observed against *Cryptococcus neoformans* and *Trichophyton interdigitalis*.

In order to quantitate the antimicrobial activity of the essential oils, the minimal inhibitory concentrations (MIC) were determined in case of selected fungal strains. The MIC values varied from 1.25 *μ*g/mL to 20.00 *μ*g/mL for the tested fungal strains (Table 3). The highest antifungal potency was exhibited by herb and root oils against *Cryptococcus neoformans* with 1.25 *μ*g/mL MIC values. In addition, substantial efficacy (MIC = 2.50 *μ*g/mL) was detected against other *Candida* strains (*C. glabrata*, *C. tropicalis*) and *Rhodotorula glutinis*.

In summary, the composition of the essential oil of the roots of *C. Canadensis* is described for the first time, with the C_10_ acetylene-type compound 2*Z*,8*Z*-matricaria ester as the main component, similarly to the closely related species *E. acris* and *E. annuus* [17]. In accordance with previous analyses [11–13], limonene was detected as the predominant component of the essential oil of aerial parts of horseweed. The antifungal activity of the essential oil of aerial parts has been investigated previously against various phytopathogenic fungi [12]; however our experiments provide the first information about the marked antifungal activity against human pathogenic strains in vitro. Further studies are needed to evaluate the in vivo potential of these oils in animal models.

## Figures and Tables

**Table 1:** Chemical composition of essential oils from herbs and roots of *Coryza canadensis. *

Compounds^a^	% in samples			RI^b^	Identification
	Herbs	Roots 1	Roots 2		
2*E*-hexanal	0.1			873	c
*α*-pinene	0.3			936	c, d
Sabinene	tr			978	c, d
*β*-pinene	2.8	1.3		985	c, d
Myrcene	1.5			992	c
*p*-cymene	tr			1020	c, d
**limonene**	**79.2**	**1.0**		1038	c, d
*trans*-ocimene	0.9			1050	c
2,5-dimethyl styrene	0.1			1101	c
*E,E-*cosmene	0.4			1132	c
*cis*-verbenol	0.5			1142	c
*trans*-sabinol	0.4			1147	c
2-allyl-phenol	0.2			1199	c
Myrtenol	0.3			1208	c
*cis*-p-mentha-1(7),8-dien-2-ol	0.8			1232	c
*trans*-chrysanthenyl acetate	0.3			1245	c
Modheph-2-ene	0.1			1389	c
*β*-caryophyllene	0.2			1427	c, d
*α*-*trans*-bergamotene	2.9	tr		1439	c
*α*-curcumene +amorpha-4,7(11)-diene	1.8			1488	c
11-*α*H-himachala-1,4-diene	0.2			1495	c
*4E,8Z*-matricaria lactone		0.7		1490	c, d
*δ*-cadinene	0.2			1526	
*8Z*-2,3-dihydromatricaria ester	1.0	3.3	2.6	1537	c
*2E,8Z*-matricaria ester	0.3	1.9	0.9	1540	c
***2Z,8Z*-matricaria ester**	**2.1**	**88.2**	**93.9**	1551	c
*4Z,8Z*-matricaria lactone	tr	2.3	2.5	1560	c
Germacrene B	0.2			1567	c
*2E,8E*-matricaria ester	—	0.7		1597	c
Spathulenol	0.3			1581	
ar-turmerone	0.1			1590	c
*β*-copaen-4-*α*-ol	0.4			1595	c
Salvia-4(14)-en-1-one	0.2			1610	c

Total	98.0	98.6	99.9		

^a^Compounds listed in sequence of elution from a CB-5 MS column.

^b^Kovats retention indices calculated against C_9_ to C_24_ *n*-alkanes on a CB-5 MS column.

^c^Comparison of mass spectra with MS libraries and retention indices.

^d^Comparison with authentic compound.

**Table 2:** Antimicrobial activities of the essential oils of *Conyza canadensis* using agar disc diffusion method.^a^

Microorganisms	Essential oil		Antibiotics		
	Herbs (8 *μ*g)	Roots (8 *μ*g)	Piperacillin/tazobactam (100/10 *μ*g)	Fluconazole (25 *μ*g)	Voriconazole (1 *μ*g)
*Enterococcus faecalis*	NA	NA	25	NT	ND
*Staphylococcus aureus*	NA	NA	30	NT	ND
*Streptococcus pyogenes*	NA	NA	35	NT	ND
*Escherichia coli*	NA	NA	25	NT	ND
*Pseudomonas aeruginosa*	NA	NA	30	NT	ND
*Candida albicans*	15	20	NT	30	40
*Candida glabrata*	25	25	NT	NA	NA
*Candida kefyr*	7	9	NT	50	50
*Candida* *parapsilosis *	13	18	NT	28	30
*Candida tropicalis*	15	18	NT	18	12
*Cryptococcus neoformans*	40	32	NT	25	35
*Rhodotorula glutinis*	13	18	NT	30	30
*Trichophyton interdigitalis*	40	40	NT	NA	> 40
*Aspergillus fumigatus*	NA	NA	NT	NA	18

^a^Diameter of inhibition zone (mm) including diameter of the paper disc (6 mm).

NA: not active.

NT: not tested.

**Table 3:** MIC determination of plant essential oils on selected fungal strains.

Microorganisms	Essential oils		Fluconazole *μ*g/mL	DMSO
	Herbs *μ*g/mL	Roots 1 *μ*g/mL		
*Candida albicans*	5.00	10.00	0.195	>20
*Candida glabrata*	2.50	2.50	NA	>20
*Candida kefyr*	10.00	20.00	0.195	>20
*Candida parapsilosis*	10.00	10.00	0.39	>20
*Candida tropicalis*	2.50	2.50	50.00	>20
*Cryptococcus neoformans*	1.25	1.25	25.00	>20
*Rhodotorula glutinis*	2.50	2.50	25.00	>20

NA: not active.
